# Global soil moisture dynamics: attribution of contributions and their association with GPP

**DOI:** 10.3389/fpls.2025.1691082

**Published:** 2025-10-31

**Authors:** Yang Li, Leer Bao, Panxing He, Chengyun Wang, Songyan Zhu, Jun Ma, Yunjiang Liang, Jianhua Xiao

**Affiliations:** 1 College of Agriculture, Yanbian University, Yanji, Jilin, China; 2 Key Laboratory of Ecological Safety and Sustainable Development in Arid Lands, Northwest Institute of Eco-Environment and Resources, Chinese Academy of Sciences, Lanzhou, Gansu, China; 3 State Key Laboratory of Wetland Conservation and Restoration, Ministry of Education Key Laboratory for Biodiversity Science and Ecological Engineering, School of Life Sciences, Fudan University, Shanghai, China; 4 State Key Laboratory of Water Resources Engineering and Management, Wuhan University, Wuhan, Hubei, China; 5 School of Geography and Environmental Sciences, University of Southampton, Southampton, England, United Kingdom

**Keywords:** SM, spatiotemporal evolution, contribution attribution, trend, IAV, GPP

## Abstract

Soil moisture (SM) is central to the global land-atmosphere interaction and is of significant research importance. However, the dynamic structural features of SM remain insufficiently explored. This study utilizes GLDAS-Noah SM data from 1948 to 2024 to develop a collaborative framework for quantifying SM contributions, based on “three-dimensional decomposition + covariance attribution.” It decomposes Total Soil Moisture Variability (TSMV) into two major temporal dynamics: long-term trends (Trend) and inter-annual variability (IAV), assessing the contributions of different soil depths, seasonal variations, and temporal dynamics to TSMV, thereby laying the methodological groundwork for analyzing global TSMV structural features. Furthermore, the relationship between SM and the gross primary productivity (GPP) of different ecosystems remains unclear. This study further integrates the MODIS MCD12C1 and GOSIF GPP datasets to explore the correlation between SM and GPP on a global scale. The results indicate that between 2000 and 2024, global Total Soil Moisture (TSM) shows a marked declining trend, with SM decreasing synchronously across all seasons. The IAV contribution from the 40–200 cm soil layer to TSMV is more significant, and this contribution exhibits notable spatial variation. Globally, SM and GPP show an overall positive correlation, particularly in the 10–100 cm root zone of grasslands and croplands, where the correlation is especially pronounced.

## 1 Introduction

Global warming, through both direct warming and water cycle feedbacks, has profoundly altered soil moisture (SM), a fundamental land–atmosphere interaction variable (Xu et al., 2013). As a regulator of the water–carbon cycle, agriculture, and climate systems, SM has long been central to global change studies (Seneviratne et al., 2010; Hao et al., 2025). Yet, widespread declines have been observed in recent decades. In East Asia (1948–2010), SM decreased significantly at –0.000645 m³/m³/10y, with severe drying in Northeast and North China, Mongolia, and near Lake Baikal (Cheng et al., 2015). The Himalaya–Tibetan Plateau (HTP) has shown persistent SM loss since the 21st century, projected to accelerate to –0.372 kg/m²/10a after 2080 under RCP8.5 (Zhang et al., 2019). In the Pearl River Basin, autumn SM declined markedly, while spring and autumn decreases dominate in North and Northwest China (Zhang et al., 2018; Han et al., 2020). Extreme events and land-use change have further amplified SM heterogeneity, driven by multi-scale nonlinear mechanisms: autumn drying in the Pearl River Basin reflects both reduced rainfall and higher temperatures, whereas winter is mainly warming-driven (Zhang et al., 2018); in East Asia, reduced precipitation dominates SM drying, but warming nearly doubles the effect (Cheng et al., 2015); on the HTP, precipitation gains are offset by 2–3-fold larger increases in potential evapotranspiration, intensifying aridification (Zhang et al., 2019). These shifts impair ecosystem functioning and, through land–atmosphere feedbacks (e.g., the North American heatwave), further accelerate drying, highlighting the urgency of understanding SM dynamics (Liu et al., 2020; 2021 North American heatwave amplified by climate change-driven nonlinear interactions; Vereecken et al., 2022).

Advances in remote sensing and monitoring have confirmed that SM variability arises from coupled climate, soil, and human influences (Lathuillière et al., 2016; Xu et al., 2019; Chen et al., 2020). Yet, current work rarely quantifies the structural features of SM variability, particularly across depth and season (Loew and Schlenz, 2011; Lee and Kim, 2017). Many studies emphasize surface SM (0–10 cm), overlooking the pivotal role of deep SM (40–100 cm) in water storage and drought recovery. This deep-layer contribution to TSM is especially critical in regions such as the HTP and arid zones (Zhang et al., 2019). In the Pearl River Basin, seasonal spatial differences in SM have been mapped (e.g., east–west gradients in spring; high east/west and low center in summer), but the contributions of different soil layers to these patterns remain unquantified (Zhang et al., 2018). Moreover, most research has focused on external climatic drivers (precipitation, circulation), without decomposing the variance structure of TSMV or assessing how soil depth and season shape Trend and IAV contributions (Cheng et al., 2015; Feng and Liu, 2015; Cheng and Huang, 2016; Wu et al., 2020). SM dynamics also show regional contrasts: warming may lower SM in drylands but increase it in humid zones (Feng and Zhang, 2015; Seddon et al., 2016; Hu et al., 2021). A multidimensional analysis of structural contributions to TSMV and their spatiotemporal expressions is urgently needed to disentangle ecosystem- and region-specific patterns.

Unlike near-surface atmospheric humidity, SM exhibits vertical stratification (Wei et al., 2025). While surface SM primarily tracks short-term climatic fluctuations, deeper SM regulates water storage and drought recovery, functions especially critical in arid systems (Wen et al., 2024). Yet, many investigations have focused narrowly on surface layers, thereby underestimating the systemic significance of deeper SM (Traff et al., 2015; Li et al., 2024). This bias limits robust evaluation of how soil-layer dynamics differentially affect Total Soil Moisture (TSM) trends and variability. Seasonal segmentation is equally indispensable for decoding SM cycles (Renninger et al., 2010; Wang et al., 2018). In A-S, heightened evaporation and diminished precipitation sustain SM deficits, whereas H-S replenishes water and stabilizes ecosystem functioning. These seasonal transitions strongly regulate terrestrial carbon fluxes, but multi-layer seasonal contributions remain underexplored. Elucidating such synergistic dynamics is thus crucial for refining hydrological models and guiding ecosystem management.

Conventional methods face challenges in disentangling temporal dynamics of SM variations across soil depths and seasons and their relative roles in TSMV. Here, using GLDAS-Noah SM data, we establish a “3D decomposition + covariance attribution” framework (Zhou et al., 2017) to quantify coordinated SM variability across depths and seasons and its contributions to TSMV. Coupled with covariance decomposition, this approach isolates the dominant drivers of TSMV. Notably, the framework innovatively applies linear regression to separate TSMV into Trend and IAV components (Equations 1–3), offering a novel strategy for quantifying the structural contributions to global TSMV.

Gross Primary Productivity (GPP), an indicator of plant capacity to convert solar energy into organic matter, is directly constrained by SM supply (Peng et al., 2024). Through its control of root water uptake and stomatal conductance, SM governs photosynthetic carbon assimilation (Li et al., 2020). Ecosystem-specific differences in rooting architecture and water-use strategies yield divergent responses to SM shifts. Yet, global-scale analyses linking SM stratification and seasonal dynamics with GPP remain limited. We address this gap by systematically quantifying SM–GPP relationships, thereby informing ecosystem vulnerability evaluations.

In summary, this work seeks to resolve the spatiotemporal dynamics of global SM, assess how soil depth, seasonal cycles, and temporal features contribute to TSMV, and examine its linkages with ecosystem-level GPP. Through this, we aim to advance scientific understanding of global SM variability and its ecological response mechanisms.

## 2 Data and methods

### 2.1 Dataset description and selection basis

To comprehensively resolve the spatiotemporal characteristics and dynamic contributions of global SM, and to evaluate its effects on ecosystem GPP under different land-cover contexts, we employed three key datasets:

The GLDAS (Global Land Data Assimilation System)–Noah Land Surface Model L4 monthly 1.0° × 1.0° degree V2.0 (1948–1999) and V2.1 (2000–2024) datasets (Rodell et al., 2004), developed by NASA, provide SM data across four depth layers (0–10 cm, 10–40 cm, 40–100 cm, and 100–200 cm). With monthly temporal resolution and global coverage, the 1.0° spatial resolution balances detail with computational efficiency for large-scale studies. GLDAS integrates multi-source observations to deliver consistent 0–200 cm SM data since 1948. Independent evaluations have demonstrated strong agreement between GLDAS-Noah and GRACE gravimetry as well as ground hydrological networks, though uncertainties remain in high-latitude and hyper-arid regions (Gou and Soja, 2024). Its reliability surpasses most comparable products and it has been widely applied in global hydrology and climate change research (Wang et al., 2016; Yang and Zhang, 2018), providing robust support for multi-layer and cross-season SM attribution analyses. To examine seasonal SM dynamics, we classified the 12 months into four categories based on each year’s monthly mean SM: the wettest three months as Humid Season (H-S), the next wettest as Sub-Humid Season (SH-S), the next driest as Semi-Arid Season (SA-S), and the driest three months as Arid Season (A-S). This classification framework balances regional heterogeneity at the global scale and facilitates unified analyses of seasonal SM dynamics.

The MODIS (Moderate Resolution Imaging Spectroradiometer) MCD12C1 land-cover dataset, derived from Terra and Aqua MODIS imagery using supervised classification algorithms, provides global land-cover information. Based on the International Geosphere–Biosphere Program (IGBP) classification system, it offers 0.05° resolution and distinguishes 17 land-cover types. Calibrated with MODIS remote sensing and ground observations, this dataset ensures classification reliability and provides essential support for land-cover analysis in this study (Friedl et al., 2010).

The GOSIF (Global OCO-2 SIF) GPP dataset is constructed from solar-induced chlorophyll fluorescence (SIF) observations acquired by the Orbiting Carbon Observatory-2 (OCO-2) (Li and Xiao, 2019a). Using a data-driven approach, it integrates OCO-2 SIF, MODIS remote sensing, and meteorological reanalysis to generate global GPP estimates with 0.05° spatial resolution, 8-day temporal resolution, and coverage from 2000–2024. Validated against GPP observations from 91 FLUXNET sites, the dataset shows strong correlations (R² = 0.73, p< 0.001), effectively capturing GPP seasonal dynamics. It has significant value for studying global GPP variability and ecosystem dynamics (Li and Xiao, 2019b).

### 2.2 Methodology

We extracted SM data for four representative soil layers (0–10 cm, 10–40 cm, 40–100 cm, 100–200 cm) from the GLDAS-Noah dataset. Soil water content (kg·m^-^²) was calculated as soil layer thickness (m) × water density (~1000 kg·m^-^³). Based on GLDAS-Noah’s layer structure (0–0.1 m, 0.1–0.4 m, 0.4–1.0 m, 1.0–2.0 m), the theoretical maximum capacities were about 100, 300, 600, and 1000 kg·m^-^², respectively. To filter unrealistic values, thresholds were set to 99.9, 299.9, 599.9, and 999 kg·m^-^² (slightly below these limits to avoid floating-point artifacts). Values above the thresholds were marked as missing (NaN) during preprocessing. This quality-control step removed obvious nonphysical anomalies and safeguarded the reliability of subsequent statistical analyses. For long-term trend analysis, the preprocessed SM was aggregated annually by computing mean values for each layer and summing across all four layers to derive TSM, representing annual soil-profile dynamics. Trend detection employed Sen’s slope estimator combined with the Mann–Kendall test (Gocic and Trajkovic, 2013), a distribution-free and outlier-robust method widely applied in climate research, enabling robust identification of long-term SM trends globally.

To quantify the contributions of soil depth and seasonality to TSMV, we applied an integrated attribution framework combining variance decomposition with regression analysis (He et al., 2022; Ma et al., 2022). The core principle is to partition SM variability into Trend and IAV components using regression, followed by variance decomposition to assess the contributions of depth and seasonal factors (see Sections 2.3–2.4).

For SM–GPP correlation analysis, we first preprocessed the 17 land-cover categories (codes 0–16) in the MCD12C1 dataset. The “Majority_Land_Cover_Type_1” layer, which encodes land-cover types as integers, was extracted, and non-vegetated/artificial surfaces were excluded by assigning NaN to Water Bodies (0), Urban and Built-up Lands (13), Permanent Snow and Ice (15), and Barren (16). Based on ecological similarity and study objectives, the remaining 11 IGBP types were regrouped into four major categories:

- Forests (Class 1): combining Evergreen Needleleaf Forests (1), Evergreen Broadleaf Forests (2), Deciduous Needleleaf Forests (3), Deciduous Broadleaf Forests (4), and Mixed Forests (5).
- Shrublands (Class 2): including Closed Shrublands (6), Open Shrublands (7), Woody Savannas (8), Savannas (9), and Permanent Wetlands (11).
- Grasslands (Class 3): corresponding to Grasslands (10).
- Croplands (Class 4): comprising Croplands (12) and Cropland/Natural Vegetation Mosaics (14).

To match the spatial resolution of GLDAS SM, a block-mode resampling approach was applied: the original 0.05° × 0.05° data were aggregated to 1° × 1° grids using 20 × 20 pixel blocks, with the mode within each block representing land cover. Binary masks were generated for each of the four land-cover classes (target = 1, others = NaN). This classification optimization enhanced dataset specificity and provided a robust basis for analyzing SM–GPP relationships across land-use types. Finally, we used Pearson’s correlation coefficient to assess the relationships between seasonal multi-layer SM from GLDAS and GPP. This coefficient quantifies linear dependence by normalizing covariance with the product of standard deviations.

### 2.3 Decomposition of SM time series into trend and IAV

To elucidate the spatiotemporal structure and drivers of global TSMV, we developed a three-dimensional attribution framework integrating seasonality, soil depth, and temporal variability (Trend and IAV). This decomposition represents a structural simplification of dominant drivers and is well-suited for characterizing the overall behavior of TSMV (Zhou et al., 2017). SM variability at each grid cell was partitioned into two components: a long-term trend (Trend) and interannual variability (IAV). For each grid cell, raw SM data were stratified by four seasons (s) and four soil depths (d), and Trend and IAV were independently fitted for each subgroup, as expressed by:

(1) $$SM_{s,d}\left(t\right)=\alpha_{s,d}\left(t\right)+\beta_{s,d}\left(t\right)$$

$SM_{s,d}\left(t\right)$: SM value at time *t*, season *s*, and soil depth *d* $\alpha_{s,d}\left(t\right)$: the Trend component for a given season *s* and depth *d*, defined within each subgroup by a linear regression model:

(2) $$\alpha_{s,d}\left(t\right)=k_{s,d}\cdot t+b$$

where $k_{s,d}\cdot t$ is the regression coefficient and *b* is the intercept; $\beta_{s,d}\left(t\right)$: the IAV component, defined as the residual between raw SM and the fitted Trend for season *s* and depth *d*:

(3) $$\beta_{s,d}\left(t\right)=SM_{s,d}\left(t\right)-\alpha_{s,d}\left(t\right)$$

### 2.4 Covariance decomposition and relative contribution estimation

To quantify the contributions of soil depth and seasonal factors to SM variability more precisely, we utilized a variance partitioning method that decomposes the total variance Var(Δϕ) of SM changes into the sum of several covariances. Under the assumption that SM variability is jointly influenced by soil depth and seasonality, the total variance was partitioned into these four principal terms:

In univariate regression, the Trend component (α) and IAV component (β) are independent (covariance = 0). Thus, the covariance between TSM  and $SM_{s,d}$, $Cov\left(TSM,SM_{s,d}\right)$, can be expressed as:

(4) $$Cov\left(TSM,SM_{s,d}\right)=Cov\left[\left(\alpha +\beta \right),\left(\alpha_{s,d}+\beta_{s,d}\right)\right]=Cov\left(\alpha ,\alpha_{s,d}\right)+Cov\left(\alpha ,\beta_{s,d}\right)+Cov\left(\beta ,\alpha_{s,d}\right)+Cov\left(\beta ,\beta_{s,d}\right)=Cov\left(\alpha ,\alpha_{s,d}\right)+Cov\left(\beta ,\beta_{s,d}\right)$$

Following the principle of variance decomposition, the total variance of SM, Var(TSM), can be partitioned into 32 covariance terms:

(5) $$Var\left(TSM\right)=Var\left(\alpha \right)+Var\left(\beta \right)=\sum_{s=1}^{4}\sum_{d=1}^{4}Cov\left(\alpha ,\alpha_{s,d}\right)+\sum_{s=1}^{4}\sum_{d=1}^{4}Cov\left(\beta ,\beta_{s,d}\right)=\sum_{s=1}^{4}\sum_{d=1}^{4}\left[Cov\left(\alpha ,\alpha_{s,d}\right)+Cov\left(\beta ,\beta_{s,d}\right)\right]=1$$

In the formula, Var(α) represents the contribution of the long-term trend (α) across the four seasons *s* and four soil depths *d* to TSMV, while Var(β)  represents the contribution of the IAV component (β) to TSMV. The 32 terms refer to the subgroups formed by the combinations of four seasons *s* and four soil depths *d*, representing the variance contributions of these subgroups to TSMV in both Trend and IAV dimensions.

Based on the ratio of the 32 covariance terms to Var(TSM), the relative contributions to TSMV can be calculated. Taking the IAV component at 0–10 cm in H-S as an example, the Relative Contribution (RC) is:

(6) $$RC_{s1,d1}^{IAV}=\frac{Cov\left(\beta ,\beta_{s1,d1}\right)}{Var\left(TSM\right)}$$

Relative Contribution of the IAV Component to TSMV in H-S:

(7) $$RC_{s1}^{IAV}=RC_{s1,d1}^{IAV}+RC_{s1,d2}^{IAV}+RC_{s1,d3}^{IAV}+RC_{s1,d4}^{IAV}$$

The relative contribution of the 0–10 cm Trend component to TSMV is:

(8) $$RC_{d1}^{Trend}=RC_{s1,d1}^{Trend}+RC_{s2,d1}^{Trend}+RC_{s3,d1}^{Trend}+RC_{s4,d1}^{Trend}$$

The relative contribution of the 0–10 cm layer in H-S to TSMV is:

(9) $$RC_{s1,d1}=\frac{Cov\left(\alpha ,\alpha_{s1,d1}\right)}{Var\left(TSM\right)}+\frac{Cov\left(\beta ,\beta_{s1,d1}\right)}{Var\left(TSM\right)}$$

Furthermore, for each spatial grid cell, the 32 terms are ranked by contribution, and the top two dominant components (Top1/Top2) and their contributions are extracted:

(10) $$\overset{\left(k\right)}{\hat{RC}}=\underset{\left(s,d\right)}{Top-k}\left(RC_{s,d}^{\alpha }+RC_{s,d}^{\beta }\right),\;k=1,2$$

From the 4 × 4 × 2 = 32 component combinations, the top two contributors to TSMV are selected as key controlling factors, and their relative contributions and structural attributes (soil depth *d* and season *s*) are extracted.

## 3 Results

### 3.1 Spatiotemporal changes in global TSM (1948–2024)

**Figure 1:**
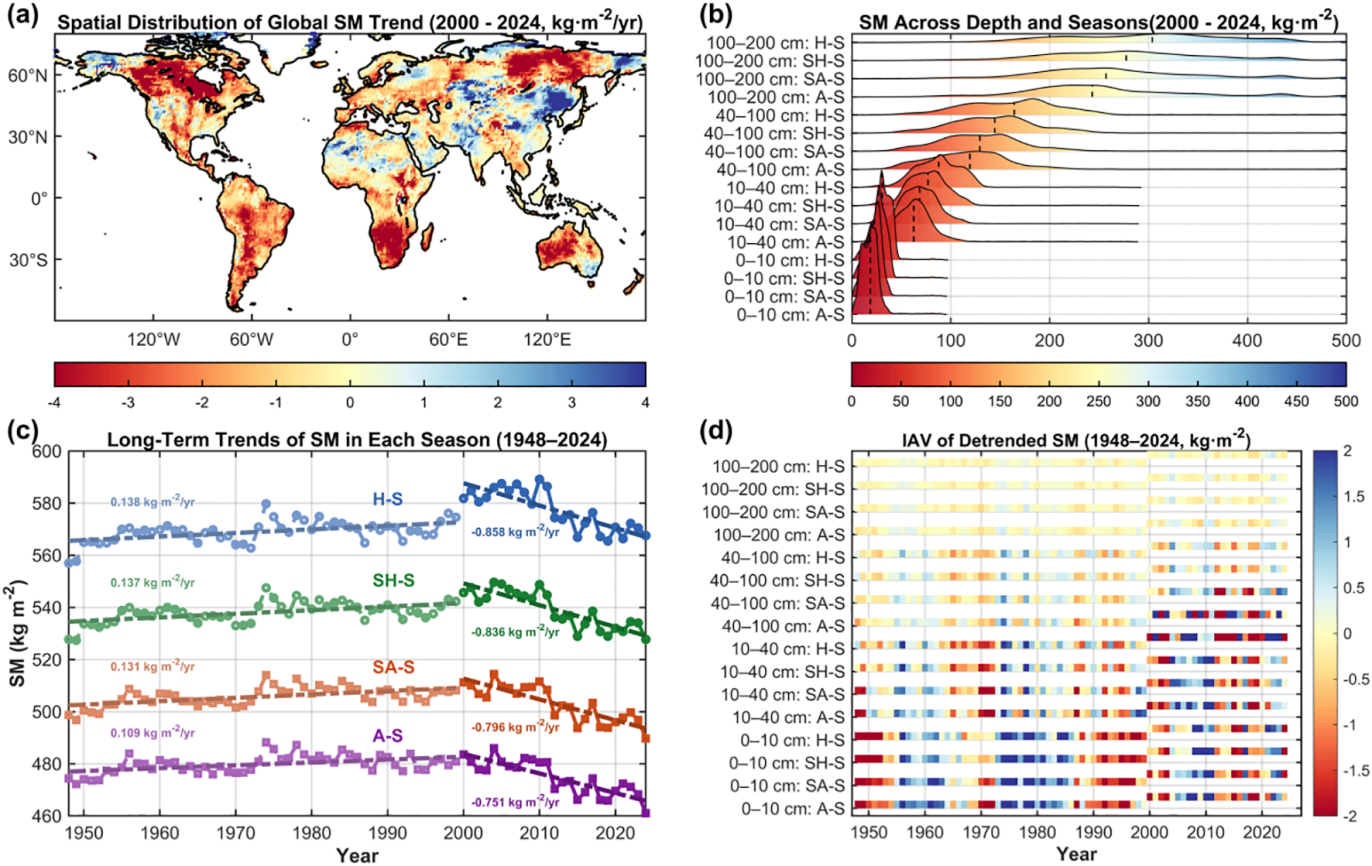
Long-term trends, vertical distribution, and IAV of global SM (1948–2024). **(a)** Spatial distribution of global SM trends for 2000–2024; **(b)** seasonal and depth-based SM distributions illustrated by ridge line color and height, with vertical dashed lines indicating mean SM; **(c)** long-term trends of seasonal mean SM during 1948–2024; **(d)** detrended IAV of seasonal and depth-resolved SM during 1948–2024, with variability represented by color gradients in heatmaps.

We first analyzed the long-term dynamics of global TSMV during 1948–2024, dividing the record into two subperiods (1948–1999 and 2000–2024) for comparison. Overall, relative to 1948–1999, global TSM exhibited a pronounced decline during 2000–2024, with a mean reduction rate of 0.87 kg·m^-^²·year^-^¹ (**Figure 1a**; **Supplementary Figure S1a**). Spatially, ~68.46% of terrestrial areas experienced declines, with 25.56% decreasing by more than 2 kg·m^-^²·year^-^¹, concentrated in Siberia, Canada, and the South African Plateau; only 7.63% of land areas showed increases >2 kg·m^-^²·year^-^¹, mainly in Northeast Asia. In contrast, during 1948–1999, TSM displayed a weak upward trend at a rate of only 0.11 kg·m^-^²·year^-^¹, with regions exceeding 2 kg·m^-^²·year^-^¹ accounting for just 1.06% (**Figure 1c**; **Supplementary Figure S1a**).

Spatial trend analysis of SM by depth for 2000–2024 revealed pronounced declines at 40–200 cm. Specifically, SM in the 100–200 cm and 40–100 cm layers declined at rates of 0.38 and 0.36 kg·m^-^²·year^-^¹, respectively (**Supplementary Figures S2a, b**); the 10–40 cm layer exhibited a milder decrease of 0.13 kg·m^-^²·year^-^¹ (**Supplementary Figure S2c**); while the 0–10 cm layer showed negligible change at 0.03 kg·m^-^²·year^-^¹ (**Supplementary Figure S2d**). Except for the surface layer, depth-specific SM trends aligned with overall TSM, with declines concentrated in North America, Siberia, and the South African Plateau. Differences in decline rates likely relate to baseline water content: the 100–200 cm layer consistently exceeded 230 kg·m^-^² across seasons, whereas the 0–10 cm layer typically remained below 35 kg·m^-^² (**Figure 1b**).

Long-term Trend diagnostics over 1948–2024 show only weak increases in seasonal SM prior to 2000 (**Figure 1c**). In contrast, after 2000, seasonal SM exhibits a sharp and significant decline (**Figures 1a,c**), closely mirroring the overall reduction in global TSM. IAV analysis further indicates consistently larger variability in the 0–10 cm layer across seasons—most pronounced in A-S—whereas the 100–200 cm layer exhibits the lowest IAV and greater stability in H-S (**Figure 1d**). Seasonally, IAV in A-S markedly exceeds that in H-S, implying higher sensitivity to short-term climate fluctuations. Collectively, global TSM has declined since 2000, while IAV persists; the 0–10 cm layer under A-S is particularly sensitive to short-term variability.

### 3.2 Spatial differentiation dominating global TSM: comparative contributions of seasonality, soil depth, and trend-IAV

**Figure 2:**
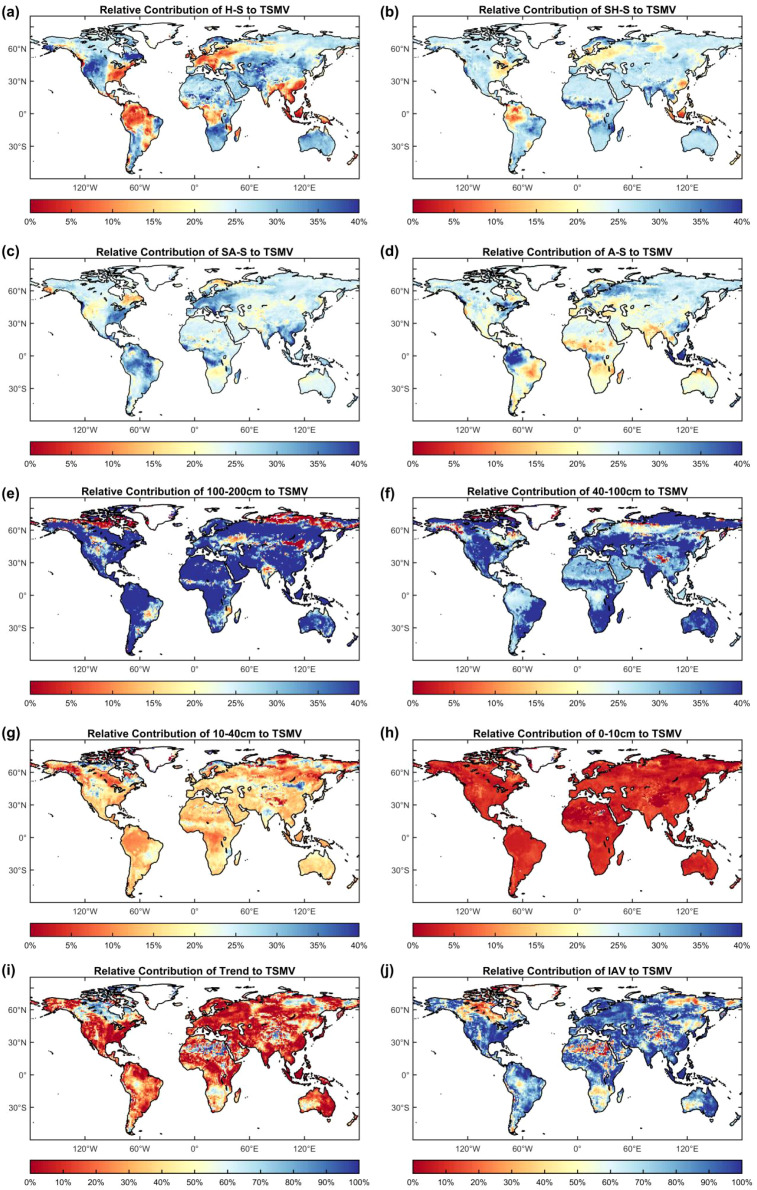
**(a–d)** spatial distributions of seasonal contributions to global pixel-wise TSMV during 2000–2024; **(e–h)** spatial distributions of contributions from soil layers; **(i, j)** relative contributions of Trend and IAV components.

Pixel-wise analysis of global TSMV relative contributions during 2000–2024 shows that seasonal components are nearly balanced, each averaging close to 25% (**Figure 2**). Specifically, H-S, SH-S, SA-S, and A-S contribute 24.30%, 25.20%, 25.57%, and 24.93%, respectively (**Figures 2a–d**). While seasonal means differ only slightly, spatial heterogeneity is pronounced, particularly across North America, Central Asia, Europe, and tropical rainforest regions. Notably, in rainforest areas, the relative contribution of H-S is typically<10%, whereas that of drier seasons often exceeds 30%.

Depth-wise comparisons indicate that SM in the 100–200 cm layer contributes most strongly to TSMV, accounting for 42.93%. Contributions decline progressively with shallower depths: 37.80% (40–100 cm), 15.27% (10–40 cm), and 4.00% (0–10 cm) (**Figures 2e–h**). Partitioning SM into long-term trend and IAV components shows IAV as the dominant driver of TSMV, contributing 75.02%, compared with only 24.98% for the trend (**Figures 2i, j**). Overall, both depth-specific and trend–IAV contributions exhibit substantial spatial heterogeneity.

### 3.3 Relative contributions of SM trend and IAV to TSMV under seasonal–depth interactions

**Figure 3:**
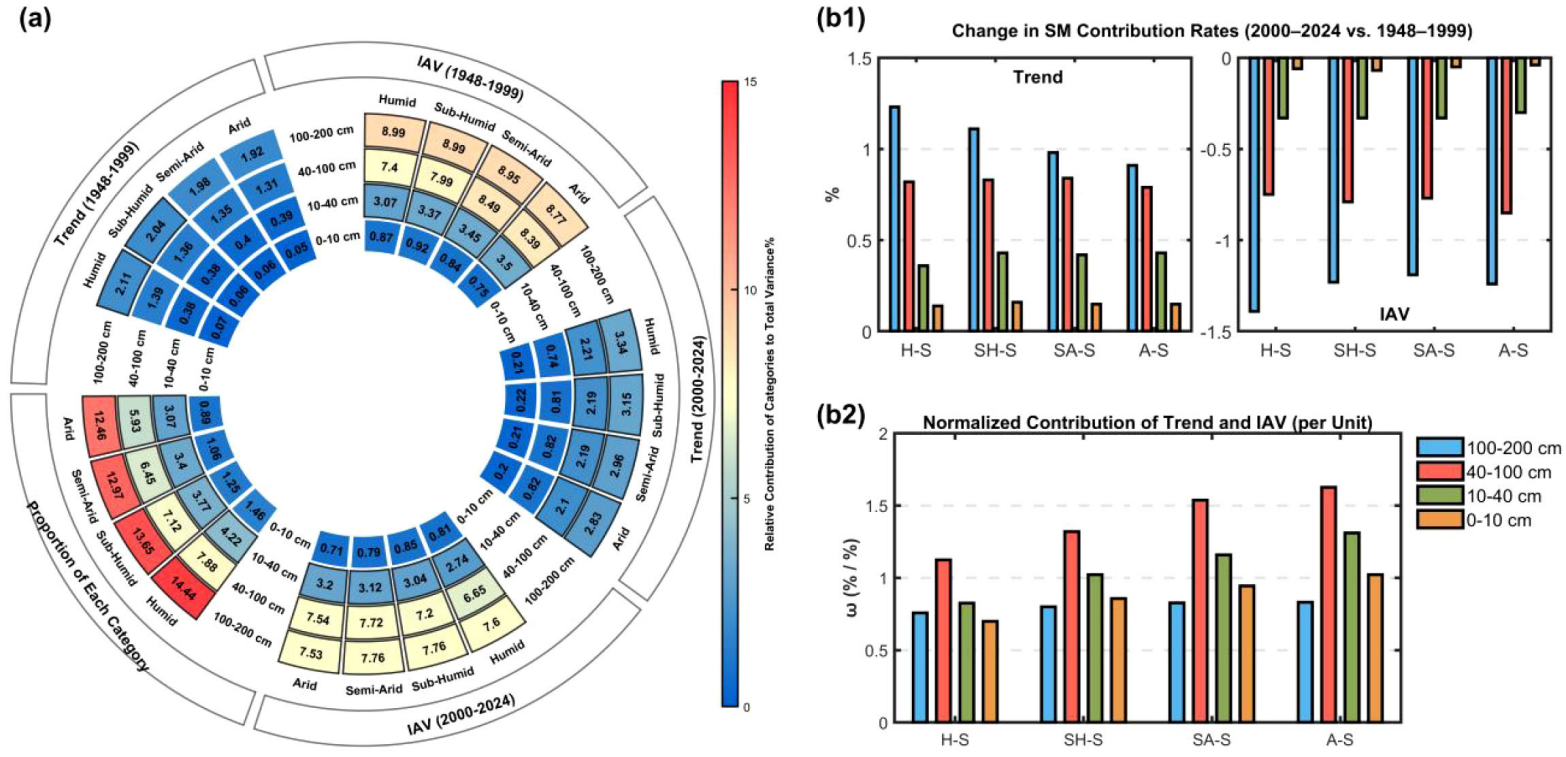
**(a)** Donut charts showing relative contributions of seasonal and depth-specific SM Trend and IAV to TSMV for 1948–2024; **(b1)** differences in seasonal and depth-specific contributions between 2000–2024 and 1948–1999; **(b2)** ω values by season and depth, defined as the sum of Trend and IAV relative contributions normalized by their proportion in TSM.

We partitioned SM data from 1948–1999 and 2000–2024 into four seasons, four soil depths, and two temporal components (IAV and Trend) to assess their contributions to TSMV (**Figure 3a**) (Equations 4–10). During 2000–2024, IAV in the 40–200 cm profile emerged as the dominant driver of TSMV, contributing >6.5% across all seasons, with the 100–200 cm IAV in SA-S and SH-S peaking at 7.76%. In 1948–1999, IAV contributions from 40–200 cm layers exceeded 7% for all seasons, with the 100–200 cm layer in H-S and SH-S reaching 8.99%.

Comparing the two periods shows an increase in Trend contributions and a decrease in IAV contributions during 2000–2024 relative to 1948–1999 (**Figure 3b1**). Depth-wise, Trend contributions vary more strongly with increasing soil depth; seasonally, wetter conditions amplify Trend shifts, with the 100–200 cm H-S component showing the largest change (+1.24%) (**Figures 3a,b1**). Moreover, differences in IAV contributions reveal that deeper soils exhibit larger inter-period changes within the same season.

Analysis of ω values across seasons and soil depths (**Figure 3b2**) indicates that within the 0–100 cm profile, ω values increase with decreasing wetness, strengthening relative contributions. The 40–100 cm layer consistently exhibits the highest ω across seasons, exceeding 1.4 in A-S and SA-S, thereby amplifying its contribution to TSMV. The 10–40 cm layer ranks second, with ω >1 in all but H-S, indicating moderate enhancement. By contrast, the 0–10 cm and 100–200 cm layers generally show ω<1, except for a slight elevation in 0–10 cm under A-S. Their contributions to TSMV thus mainly depend on share proportion, reflecting a weakening effect and underscoring depth–season contrasts in relative contributions.

### 3.4 Correlations between global TSM and ecosystem-specific GPP

**Figure 4:**
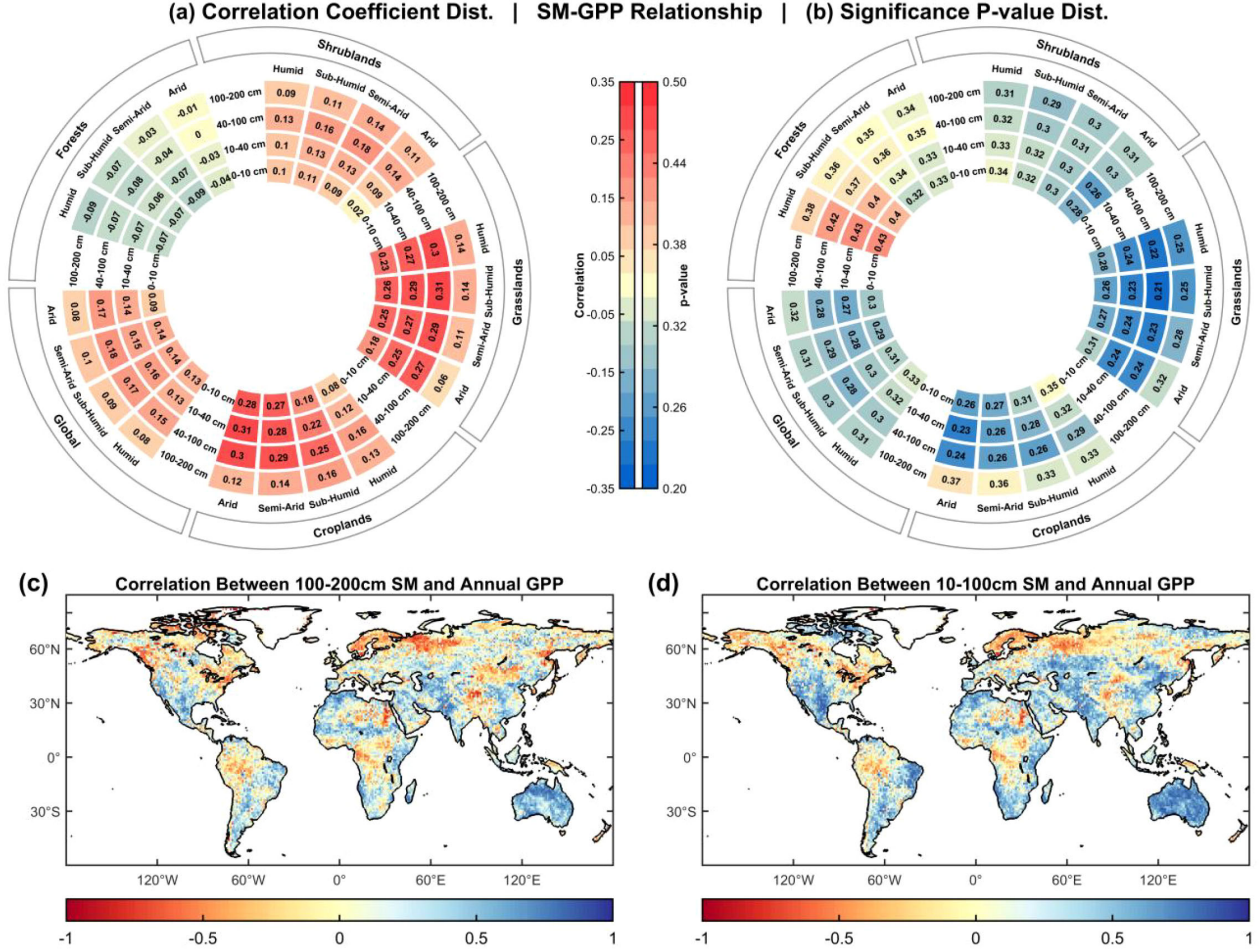
Donut plots showing SM–GPP correlations across seasons and depths for Forests, Shrublands, Grasslands, Croplands, and at the global scale. Subplots: **(a)** distribution of Pearson correlation coefficients; **(b)** distribution of significance p-values; **(c)** pixel-wise correlation of 100–200 cm SM with GPP globally; **(d)** pixel-wise correlation of 10–100 cm SM with GPP globally.

**Figure 4** evaluates the correlations and significance distributions of SM–GPP relationships across seasons, soil depths, and ecosystem types. Overall, SM and GPP are generally positively correlated, but the strength and fraction of significant regions exhibit pronounced heterogeneity across temporal, vertical, and ecological dimensions.

At the global scale, SM–GPP correlations range from 0.07 to 0.18, indicating weak overall associations but clear depth stratification and climatic dependence. Correlations are strongest in the 40–100 cm layer, peaking under SA-S (r = 0.1801), and weakest in the 100–200 cm layer, with a minimum under A-S (r = 0.0770) (**Figure 4a**). Spatially, pixel-wise correlations in the 10–100 cm layer show R > 0.2 across most regions, with the exception of high-latitude northern areas and parts of equatorial rainforests. Strong correlations are observed in the Northeast China Plain croplands, Ganges croplands, U.S. western plains, and Australia. By contrast, the 100–200 cm layer exhibits predominantly negative correlations globally, with only a few regions showing positive values (**Figures 4c,d**). Significance tests corroborate this pattern: mean p-values range from 0.28 to 0.33, with 7.1%–9.6% of pixels significant. While the global effects are modest, localized clusters of significance emerge (**Figure 4b**).

Seasonal conditions modulate these correlations. Under H-S and SH-S, SM–GPP linkages and significant fractions are relatively low (~8%). In contrast, under SA-S and A-S, correlations strengthen markedly, with the 40–100 cm and 10–40 cm layers contributing 9.1%–9.6% significant areas. In particular, the 10–40 cm layer under A-S shows the highest correlation (r = 0.1407; 9.58% significant), indicating stronger water limitation of GPP under moisture stress, especially in shallow to mid-depth layers. Despite weaker overall correlations under H-S, localized strong SM–GPP coupling persists, especially in the 100–200 cm layer, where 7.91% of pixels remain significant at p< 0.05 (**Figures 4a, b**).

Clear ecosystem-specific contrasts also emerge. In Forests, SM–GPP correlations are largely negative (r = −0.0945 to −0.0028), suggesting weak water dependence of GPP; weak but significant areas account for 9%–21%, likely reflecting deep-root architecture and nonlinear water-use strategies. Grasslands and Croplands display markedly stronger correlations, with significant areas covering 29.6% and 27.2%, respectively. Grasslands show peak correlations in the 40–100 cm layer under SH-S and H-S (r ≈ 0.31; >35% significant), whereas Croplands peak under A-S in the 10–40 cm layer (r = 0.3102; 34.78% significant) (**Figures 4a, b**). These results highlight the heightened sensitivity of Grasslands and Croplands to shallow–midroot SM. By contrast, deep soil water (100–200 cm) exerts only a weak influence on GPP in both ecosystems, with r generally<0.16.

Shrublands occupy an intermediate position, with r values of 0.08–0.18 across depths and significant areas covering 26.9%. The 10–40 cm layer under A-S is most influential, with 31.33% of pixels significant, highlighting the strong control of shallow–mid SM on photosynthesis under drought conditions.

To provide a clearer overview of the results, we constructed the Comprehensive Table of the Relationships between SM and GPP and Associated Indicators across the Globe and in Different Ecosystems. This table compiles correlation coefficients, significance levels (P-values), numbers of significant pixels, and their proportions across ecosystems, seasons, and soil depths (see **Supplementary Table**).

In summary, global SM–GPP correlations are weak on average (mostly<0.18), and mean p-values generally exceed 0.25, indicating limited overall effects. Nonetheless, the presence of localized significant clusters highlights SM’s critical regulatory role in shaping GPP at regional scales.

## 4 Discussions

### 4.1 Enhanced aridity of global SM

Analysis of GLDAS-Noah SM data indicates relatively stable conditions from 1948–1999, followed by a progressive decline since the 21st century, evident across seasons and depths. This finding aligns with previous reports: global surface SM declined significantly during 1979–2017, with drying rates accelerating after 2001 (Deng et al., 2020). Other studies similarly noted SM reductions in most regions during the 21st century, particularly across mid-latitude and tropical zones, driven by warming and intensified evapotranspiration (Zhao and Dai, 2015). Our analysis further reveals that over the past 25 years, the relative contribution of Trend to SM change increased by at least 2% compared with the late 20th century, potentially linked to a “warming amplification effect” in arid–semiarid zones, where SM depletion and vegetation loss enhance sensible heat flux and intensify land–energy imbalance (Huang et al., 2016). Seasonal SM consistently shows declining Trends. Land–atmosphere coupling experiments confirm that seasonal SM reductions largely result from internal feedbacks of seasonal SM variability on surface water availability. As the primary water source for terrestrial evapotranspiration, declining SM limits transpiration and soil evaporation, reducing regional ET, altering circulation, and enhancing moisture convergence—highlighting SM–atmosphere feedbacks as key drivers of seasonal water availability (Zhou et al., 2022). SM variability is jointly shaped by climate change and anthropogenic activity. Large-scale irrigation, land-use change, and overexploitation of water resources alter regional hydrological cycles, further intensifying SM declines in certain regions (Liu et al., 2023).

Global TSMV exhibits marked spatial heterogeneity. For example, SM declined in Canada and southern Africa, whereas slight increases were observed in Alaska and Northeast China, likely linked to greater precipitation and snowmelt inputs. Contrasting SM Trends in adjacent high-latitude regions—Canada versus Alaska—are particularly noteworthy. CLM simulations suggest that although permafrost zones receive increasing net water input (precipitation–evaporation), thaw-induced permeability enhances drainage, potentially causing widespread soil drying (Lawrence et al., 2015). In Canada, SM decreases as thaw-driven drainage offsets water gains, whereas Alaska shows SM increases where slower thaw, terrain-limited drainage, or greater recharge mitigate losses. These patterns underscore the spatial heterogeneity of permafrost impacts, governed by the balance of recharge versus drainage (Lawrence et al., 2015).

Similarly, regional SM heterogeneity across high-altitude cold zones such as the Tibetan Plateau (TP) reflects temperature-driven hydro-ecological processes. In northern TP highlands, SM increases and correlates positively with temperature, primarily due to thawing permafrost and glacier meltwater inputs (Shi et al., 2021). Conversely, in southern and southeastern TP, SM declines and correlates negatively with temperature, as warming accelerates evapotranspiration and alleviates low-temperature constraints on vegetation growth, enhancing water consumption (Shi et al., 2021). This dual mechanism of “warming-driven ET intensification + vegetation water use” complements the high-latitude permafrost “thaw–drainage” mechanism, together illustrating both shared and region-specific SM responses to climate change.

Rising ET in western North America and southern Africa has intensified soil drying, causing marked SM declines, consistent with our global TSM spatiotemporal analyses (Lal et al., 2023). Against the backdrop of global warming, SM dynamics have shifted from short-term fluctuations to persistent long-term decline, with accelerated downward Trends in the past 25 years. Projections under high-emission scenarios indicate further intensification of global TSM drying, with disproportionate impacts on drought-sensitive and agriculturally intensive regions, posing critical risks to ecosystems, food production, and water management (Salley et al., 2016; Berg et al., 2017). Satellite observations corroborate the global SM decline. For example, ESA CCI surface SM data (2000–2021) highlighted single-factor influences of land cover, soil texture, climate (precipitation/temperature), and vegetation on SM decreases, without addressing vertical heterogeneity or IAV (Peng et al., 2023; Feng et al., 2025). Our study extends this scope to the full soil profile (0–200 cm), innovatively decomposing TSMV into three dimensions—depth, season, and temporal dynamics (Trend/IAV)—and quantifying 32 intrinsic components (4 depths × 4 seasons × 2 dynamics). We also provide the first quantification of seasonal and depth-specific Trend versus IAV divergence. Future research should strengthen sensitivity analyses of regional SM changes, incorporating multi-factor synergies to improve prediction accuracy under climate change.

### 4.2 Dominant role of mid–deep SM IAV in governing TSMV

Our results show that IAV of mid–deep SM (40–200 cm) exerts primary control on TSMV during A-S. Although the 0–10 cm layer exhibits pronounced interannual and seasonal fluctuations, its limited storage constrains contributions to TSMV; by contrast, larger storage at depth allows moisture variations to dominate TSMV’s IAV (Harper et al., 2010). The effect is especially pronounced in SA-S and A-S, where deep soils act as a stabilizing reservoir that buffers shallow-layer volatility. Under A-S, when surface SM is rapidly depleted by evaporation and transpiration, mid–deep SM sustains system stability via two pathways: hydraulic lift transports deep water to the surface within dense rooting zones, easing shallow drought stress (Tong et al., 2020); and in the 100–200 cm layer, gravity-driven matric gradients promote downward–upward redistribution, creating a “cryptic water source,” consistent with deep-profile observations on the Loess Plateau (Tong et al., 2020). These findings highlight the hydrologic importance of deep SM as a steady release reservoir under drought.

Comparing 2000–2024 with 1948–1999, the contribution of mid–deep SM IAV to TSMV declined somewhat yet remained dominant. This attenuation likely reflects enhanced extraction of deep SM by intensified ET under climate change. Attribution using ERA5-Land indicates that post-2000 the ET share from mid–deep soils increased, accelerating depletion of deep storage (Jiang et al., 2022). In addition, groundwater overexploitation weakens capillary recharge to mid–deep SM—a pattern corroborated by numerical experiments for the North China Plain and elsewhere (Sun et al., 2025).

We also find pronounced vertical differentiation in IAV contributions: with depth, the influence on TSMV strengthens, consistent with the “soil reservoir effect.” In this view, deep soils act as a slow climate variable with long memory for land-surface processes, whereas shallow SM primarily tracks short-term weather fluctuations (Vereecken et al., 2022). This vertical heterogeneity is operationally salient for drought management: integrating deep-soil monitoring into regional early-warning systems can anticipate shifts in vegetation productivity (Sheffield et al., 2012).

### 4.3 Seasonal differentiation of TSMV contributions in tropical rainforests

In tropical rainforests, SM IAV shows marked seasonal asymmetry: A-S variability exceeds H-S, diverging from the global midlatitude pattern where H-S dominates. This highlights unique hydrothermal coupling and heightened sensitivity of rainforests to extreme climate. Elevated A-S variability in rainforests arises from large rainfall fluctuations coupled with strong transpiration. During El Niño events, anomalous Pacific SST shifts the ITCZ southward, sharply reducing A-S precipitation and triggering cascading SM declines (Le and Bae, 2022). Prolonged A-S further enhances SM variability: vegetation reduces stomatal conductance to limit losses, but canopy interception declines, producing a positive feedback that amplifies SM fluctuations (Barros et al., 2019). Additional drivers include climate warming, which raises potential ET, and human disturbances such as deforestation that elevate albedo, suppress rainfall, and reduce soil water-holding capacity—together intensifying SM variability (Leite-Filho et al., 2019; Leite-Filho et al., 2021; Xu et al., 2022). Satellite records reveal that in Brazil’s Amazon agricultural frontiers, A-S SM variability coefficients are markedly higher than in intact forests (Leite-Filho et al., 2021).

By contrast, rainforest H-S exhibits low SM IAV due to balanced water budgets: abundant rainfall and moderate ET maintain SM near field capacity, restricting interannual variability (Lathuillière et al., 2012; Aguilos et al., 2019). Crucially, deep-root systems (mean depth ~2.5 m) hydraulically lift deep water to the surface, stabilizing SM during rainfall gaps (Harper et al., 2010; Kühnhammer et al., 2023). In H-S, these roots also form a hydraulic redistribution network that stores excess water in aquifers, buffering subsequent A-S deficits.

Global climate change is disrupting this balance (Lynn and Peeva, 2021). In the Amazon, SM stability during dry seasons has relied on deep reservoirs (to ~10 m), hydraulic redistribution, and deep-root uptake (Baker et al., 2008; Lan et al., 2016; Huang et al., 2021). Yet evidence shows that when dry-season rainfall falls below ~100 mm per month or droughts extend, surface SM rapidly depletes and deep-soil recharge efficiency declines with drought intensity (Baker et al., 2008). This implies that if climate change increases extreme drought frequency in the Amazon, dry-season SM stability will be severely disrupted, with variability exceeding that of climatically stable periods (Baker et al., 2008; Aguilos et al., 2018). The seasonal asymmetry of SM variability identified here offers new insights into land-surface processes under global change.

### 4.4 Vertical differentiation of global SM–GPP relationships

Intensifying human activity is amplifying the influence of SM on GPP (Ren et al., 2025). Although deep SM (100–200 cm) contributes substantially to TSMV, its correlation with GPP is limited. By contrast, SM in the 10–100 cm zone—directly accessible to plant uptake—plays the dominant role, especially in Croplands and Grasslands, consistent with the “root-depth–productivity” paradigm (Mueller et al., 2013).

In Croplands and Grasslands, root biomass is concentrated in the 10–100 cm profile, where water uptake per unit root length is far higher than in deeper layers (Cordeiro et al., 2020; Ma et al., 2021). Thus, water availability here directly governs stomatal conductance, strongly influencing photosynthetic carbon fixation (Hu et al., 2018; Vargas Zeppetello et al., 2023). This underscores the central role of shallow–mid SM in modulating plant water stress and directly driving photosynthetic assimilation.

In contrast, deep SM (100–200 cm) influences GPP indirectly. During extreme drought, it sustains plants via lagged mechanisms such as embolism repair and capillary rise that buffer shallow–mid moisture, providing a “cryptic water source” capable of extending supply by 3–6 months (Kitajima et al., 2013; Nazarieh et al., 2018; Guo et al., 2024). Under prolonged drought, ABA signaling promotes deep-root proliferation, but requires 2–3 weeks of physiological adjustment (Cenzano et al., 2014), whereas shallow–mid roots respond more rapidly (Li et al., 2022). Thus, while deep SM aids drought resistance, its effect on GPP is delayed.

Increasing frequency of extreme drought is likely to amplify shallow–mid SM fluctuations, further strengthening the dominance of the 10–100 cm zone over GPP. Such changes may heighten ecosystem vulnerability and risk “soil–vegetation” feedback imbalances in semiarid regions (Li and Sawada, 2022). By quantifying vertical differentiation in SM–GPP linkages, our study underscores the primacy of root-zone SM for productivity and informs both SM model optimization and drought-sensitive SM management.

## 5 Conclusions

This study analyzed global TSM trends, structural contributions, and linkages with ecosystem GPP. Results show that global TSM declined significantly during 2000–2024. The 100–200 cm layer and IAV dominate TSMV, with pronounced spatial heterogeneity. Globally, SM is positively correlated with GPP, with the 40–100 cm layer showing the strongest correlation, particularly in Grasslands and Croplands. The novelty of this study lies in moving beyond regional analyses to, for the first time, quantify component-wise contributions to TSMV at the global scale across temporal and spatial dimensions, incorporating soil-depth and seasonal differentiation to fill a major gap in coupled TSM dynamics research.

Theoretical implications include: (i) revealing the dominant role of deep SM IAV and the rising Trend contribution to TSMV, challenging the long-held view that surface SM governs land–atmosphere exchange; (ii) establishing quantitative links between TSM dynamics in cold regions and near-surface energy fluxes (albedo shifts from permafrost thaw, latent heat adjustments from enhanced ET) and atmospheric moisture cycling (precipitation feedbacks from TSM anomalies), thereby improving land-surface models by incorporating “TSM–energy–moisture” coupling and correcting biases from neglecting cold-region heterogeneity; (iii) demonstrating vertical and seasonal differentiation in SM–GPP linkages, providing global-scale evidence of water–carbon coordination within the soil–plant–atmosphere continuum (SPAC).

Practically, this work offers a theoretical foundation for ecosystem management, with implications for agriculture, water-resource governance, and climate-change adaptation. Limitations include insufficient spatiotemporal resolution of datasets, constraining representation of fine-scale processes and lowering precision in quantifying mechanisms such as recharge–drainage balance or ET–vegetation coupling. Moreover, the framework does not encompass all possible complex interactions. Future directions include: acquiring high-resolution long-term datasets to isolate small-scale drivers masked in large-scale simulations, improving attribution of recharge–drainage and ET–vegetation feedbacks; refining methods and conducting deeper regional sensitivity analyses; and ultimately enhancing TSMV predictive accuracy to support global ecological sustainability.

## Data Availability

The original contributions presented in the study are included in the article/**Supplementary Material**. Further inquiries can be directed to the corresponding authors.

## References

[B1] Aguilos M. Hérault B. Burban B. Wagner F. Bonal D. (2018). What drives long-term variations in carbon flux and balance in a tropical rainforest in French Guiana? Agric. For. Meteorol. 253-254, 114–123. doi: 10.1016/j.agrformet.2018.02.009

[B2] Aguilos M. Stahl C. Burban B. Hérault B. Courtois E. Coste S. . (2019). Interannual and seasonal variations in ecosystem transpiration and water use efficiency in a tropical rainforest. Forests 10, 14. doi: 10.3390/f10010014

[B3] 2021 North American heatwave amplified by climate change-driven nonlinear interactions . Nat. Climate Change 12, 1096–1097. doi: 10.1038/s41558-022-01532-0

[B4] Baker I. T. Prihodko L. Denning A. S. Goulden M. Miller S. da Rocha H. R. (2008). Seasonal drought stress in the Amazon: Reconciling models and observations. J. Geophys. Res.: Biogeosci. 113, G00B01. doi: 10.1029/2007JG000644

[B5] Barros F. Bittencourt P. R. L. Brum M. Restrepo-Coupe N. Pereira L. Teodoro G. S. . (2019). Hydraulic traits explain differential responses of Amazonian forests to the 2015 El Niño-induced drought. New Phytol. 223, 1253–1266. doi: 10.1111/nph.15909, PMID: 31077396

[B6] Berg A. Sheffield J. Milly P. C. D. (2017). Divergent surface and total soil moisture projections under global warming. Geophys. Res. Lett. 44, 236–244. doi: 10.1002/2016GL071921

[B7] Cenzano A. M. Masciarelli O. Luna M. V. (2014). Abscisic acid metabolite profiling as indicators of plastic responses to drought in grasses from arid Patagonian Monte (Argentina). Plant Physiol. Biochem. 83, 200–206. doi: 10.1016/j.plaphy.2014.07.024, PMID: 25245790

[B8] Chen D. Wei W. Chen L. (2020). How can terracing impact on soil moisture variation in China? A meta-analysis. Agric. Water Manage. 227, 105849. doi: 10.1016/j.agwat.2019.105849

[B9] Cheng S. Guan X. Huang J. Ji F. Guo R. (2015). Long-term trend and variability of soil moisture over East Asia. J. Geophys. Res.: Atmospheres. 120, 8658–8670. doi: 10.1002/2015JD023206

[B10] Cheng S. Huang J. (2016). Enhanced soil moisture drying in transitional regions under a warming climate. J. Geophys. Res.: Atmospheres. 121, 2542–2555. doi: 10.1002/2015JD024559

[B11] Cordeiro A. L. Norby R. J. Andersen K. M. Valverde-Barrantes O. Fuchslueger L. Oblitas E. . (2020). Fine-root dynamics vary with soil depth and precipitation in a low-nutrient tropical forest in the Central Amazonia. Plant-Environ. Interact. 1, 3–16. doi: 10.1002/pei3.10010, PMID: 37284129 PMC10168058

[B12] Deng Y. Wang S. Bai X. Luo G. Wu L. Cao Y. . (2020). Variation trend of global soil moisture and its cause analysis. Ecol. Indic. 110, 105939. doi: 10.1016/j.ecolind.2019.105939

[B13] Feng H. Liu Y. (2015). Combined effects of precipitation and air temperature on soil moisture in different land covers in a humid basin. J. Hydrol. 531, 1129–1140. doi: 10.1016/j.jhydrol.2015.11.016

[B14] Feng H. H. Wang S. Li S. J. Wang W. Li J. Y. Gu R. X. . (2025). Satellite-based re-examination of global soil moisture variation. Adv. Space. Res. 75, 3486–3495. doi: 10.1016/j.asr.2024.12.030

[B15] Feng H. Zhang M. (2015). Global land moisture trends: drier in dry and wetter in wet over land. Sci. Rep. 5, 18018. doi: 10.1038/srep18018, PMID: 26658146 PMC4676011

[B16] Friedl M. A. Sulla-Menashe D. Tan B. Schneider A. Ramankutty N. Sibley A. . (2010). MODIS Collection 5 global land cover: Algorithm refinements and characterization of new datasets. Remote Sens. Environ. 114, 168–182. doi: 10.1016/j.rse.2009.08.016

[B17] Gocic M. Trajkovic S. (2013). Analysis of changes in meteorological variables using Mann-Kendall and Sen’s slope estimator statistical tests in Serbia. Global Planet. Change 100, 172–182. doi: 10.1016/j.gloplacha.2012.10.014

[B18] Gou J. Soja B. (2024). Global high-resolution total water storage anomalies from self-supervised data assimilation using deep learning algorithms. Nat. Water 2, 139–150. doi: 10.1038/s44221-024-00194-w

[B19] Guo J.-J. Gong X.-W. Li X.-H. Zhang C. Duan C.-Y. Lohbeck M. . (2024). Coupled hydraulics and carbon economy underlie age-related growth decline and revitalisation of sand-fixing shrubs after crown removal. Plant. Cell Environ. 47, 2999–3014. doi: 10.1111/pce.14923, PMID: 38644635

[B20] Han G. Wang J. Pan Y. Huang N. Zhang Z. Peng R. . (2020). Temporal and spatial variation of soil moisture and its possible impact on regional air temperature in China. Water 12, 1807. doi: 10.3390/w12061807

[B21] Hao Y. Mao J. Bachmann C. M. Hoffman F. M. Koren G. Chen H. . (2025). Soil moisture controls over carbon sequestration and greenhouse gas emissions: a review. NPJ Climate Atmospheric. Sci. 8, 16. doi: 10.1038/s41612-024-00888-8

[B22] Harper A. B. Denning A. S. Baker I. T. Branson M. D. Prihodko L. Randall D. A. (2010). Role of deep soil moisture in modulating climate in the Amazon rainforest. Geophys. Res. Lett. 37, L05802. doi: 10.1029/2009GL042302

[B23] He P. Ma X. Sun Z. (2022). Interannual variability in summer climate change controls GPP long-term changes. Environ. Res. 212, 113409. doi: 10.1016/j.envres.2022.113409, PMID: 35523276

[B24] Hu Z. Shi H. Cheng K. Zhou J. Pan Y. (2018). Joint structural and physiological control on the interannual variation in productivity in a temperate grassland: A data-model comparison. Global Change Biol. 24, 2965–2979. doi: 10.1111/gcb.14274, PMID: 29665249

[B25] Hu B. Wang L. Li X. Wang Y.-P. Piao S. Li Y. . (2021). Divergent changes in terrestrial water storage across global arid and humid basins. Geophys. Res. Lett. 48, e2020GL091069. doi: 10.1029/2020GL091069

[B26] Huang H. Cui H. Ge Q. (2021). Assessment of potential risks induced by increasing extreme precipitation under climate change. Natural Hazards. 108, 2059–2079. doi: 10.1007/s11069-021-04768-9

[B27] Huang J. Yu H. Guan X. Guo R. (2016). Accelerated dryland expansion under climate change. Nat. Climate Change 6, 166–171. doi: 10.1038/nclimate2837

[B28] Jiang K. Pan Z. Pan F. Wang J. Han G. Song Y. . (2022). Influence patterns of soil moisture change on surface-air temperature difference under different climatic background. Sci. Total. Environ. 822, 153607. doi: 10.1016/j.scitotenv.2022.153607, PMID: 35114238

[B29] Kitajima K. Allen M. F. Goulden M. L. (2013). Contribution of hydraulically lifted deep moisture to the water budget in a Southern California mixed forest. J. Geophys. Res.: Biogeosci. 118, 1561–1572. doi: 10.1002/2012JG002255

[B30] Kühnhammer K. van Haren J. Kübert A. Bailey K. Dubbert M. Hu J. . (2023). Deep roots mitigate drought impacts on tropical trees despite limited quantitative contribution to transpiration. Sci. Total. Environ. 893, 164763. doi: 10.1016/j.scitotenv.2023.164763, PMID: 37308023 PMC10331952

[B31] Lal P. Shekhar A. Gharun M. Das N. N. (2023). Spatiotemporal evolution of global long-term patterns of soil moisture. Sci. Total. Environ. 867, 161470. doi: 10.1016/j.scitotenv.2023.161470, PMID: 36634770

[B32] Lan C.-W. Lo M.-H. Chou C. Kumar S. (2016). Terrestrial water flux responses to global warming in tropical rainforest areas. Earth’s. Future 4, 210–224. doi: 10.1002/2015EF000350

[B33] Lathuillière M. J. Coe M. T. Johnson M. S. (2016). A review of green- and blue-water resources and their trade-offs for future agricultural production in the Amazon Basin: what could irrigated agriculture mean for Amazonia? Hydrol. Earth Syst. Sci. 20, 2179–2194. doi: 10.5194/hess-20-2179-2016

[B34] Lathuillière M. J. Johnson M. S. Donner S. D. (2012). Water use by terrestrial ecosystems: temporal variability in rainforest and agricultural contributions to evapotranspiration in Mato Grosso, Brazil. Environ. Res. Lett. 7, 24024. doi: 10.1088/1748-9326/7/2/024024

[B35] Lawrence D. M. Koven C. D. Swenson S. C. Riley W. J. Slater A. G. (2015). Permafrost thaw and resulting soil moisture changes regulate projected high-latitude CO2 and CH4 emissions. Environ. Res. Lett. 10, 94011. doi: 10.1088/1748-9326/10/9/094011

[B36] Le T. Bae D.-H. (2022). Causal impacts of el niño–southern oscillation on global soil moisture over the period 2015–2100. Earth’s. Future 10, e2021EF002522. doi: 10.1029/2021EF002522

[B37] Lee E. Kim S. (2017). Pattern similarity based soil moisture analysis for three seasons on a steep hillslope. J. Hydrol. 551, 484–494. doi: 10.1016/j.jhydrol.2017.06.028

[B38] Leite-Filho A. T. de Sousa Pontes V. Y. Costa M. H. (2019). Effects of deforestation on the onset of the rainy season and the duration of dry spells in southern amazonia. J. Geophys. Res.: Atmospheres. 124, 5268–5281. doi: 10.1029/2018JD029537

[B39] Leite-Filho A. T. Soares-Filho B. S. Davis J. L. Abrahão G. M. Börner J. (2021). Deforestation reduces rainfall and agricultural revenues in the Brazilian Amazon. Nat. Commun. 12, 2591. doi: 10.1038/s41467-021-22840-7, PMID: 33972530 PMC8110785

[B40] Li Y. Li K. Zhou Q. Zhao Y. Cai L. Yang Z. . (2024). Spatiotemporal dynamics and similarity in soil moisture in shallow soils on karst slopes. J. Hydrol. 639, 131655. doi: 10.1016/j.jhydrol.2024.131655

[B41] Li W. Migliavacca M. Forkel M. Denissen J. M.C. Reichstein M. Yang H. . (2022). Widespread increasing vegetation sensitivity to soil moisture. Nat. Commun. 13, 3959. doi: 10.1038/s41467-022-31667-9, PMID: 35803919 PMC9270344

[B42] Li S. Sawada Y. (2022). Soil moisture-vegetation interaction from near-global *in-situ* soil moisture measurements. Environ. Res. Lett. 17, 114028. doi: 10.1088/1748-9326/ac9c1f

[B43] Li X. Xiao J. (2019a). Mapping photosynthesis solely from solar-induced chlorophyll fluorescence: A global, fine-resolution dataset of gross primary production derived from OCO-2. Remote Sens. 11, 2563. doi: 10.3390/rs11212563

[B44] Li X. Xiao J. (2019b). A global, 0.05-degree product of solar-induced chlorophyll fluorescence derived from OCO-2, MODIS, and reanalysis data. Remote Sens. 11. doi: 10.3390/rs11050517

[B45] Li X. Xiao J. F. Kimball J. S. Reichle R. H. Scott R. L. Litvak M. E. . (2020). Synergistic use of SMAP and OCO-2 data in assessing the responses of ecosystem productivity to the 2018 US drought. Remote Sens. Environ. 251, 112062. doi: 10.1016/j.rse.2020.112062

[B46] Liu B. Tan X. Gan T. Y. Chen X. Lin K. Lu M. . (2020). Global atmospheric moisture transport associated with precipitation extremes: Mechanisms and climate change impacts. WIREs. Water 7, e1412. doi: 10.1002/wat2.1412

[B47] Liu Y. Yang Y. Song J. (2023). Variations in global soil moisture during the past decades: climate or human causes? Water Resour. Res. 59, e2023WR034915. doi: 10.1029/2023WR034915

[B48] Loew A. Schlenz F. (2011). A dynamic approach for evaluating coarse scale satellite soil moisture products. Hydrol. Earth Syst. Sci. 15, 75–90. doi: 10.5194/hess-15-75-2011

[B49] Lynn J. Peeva N. (2021). Communications in the IPCC’s sixth assessment report cycle. Clim. Change 169, 18. doi: 10.1007/s10584-021-03233-7, PMID: 34866716 PMC8630418

[B50] Ma X. He P. Zeng Y. Ma J. Wu X. (2022). A global-drive analysis of ecosystem respiration in the Arctic and Third Pole. Ecol. Indic. 145, 109668. doi: 10.1016/j.ecolind.2022.109668

[B51] Ma H. Mo L. Crowther T. W. Maynard D. S. van den Hoogen J. Stocker B. D. . (2021). The global distribution and environmental drivers of aboveground versus belowground plant biomass. Nat. Ecol. Evol. 5, 1110–1122. doi: 10.1038/s41559-021-01485-1, PMID: 34168336

[B52] Mueller K. E. Tilman D. Fornara D. A. Hobbie S. E. (2013). Root depth distribution and the diversity–productivity relationship in a long-term grassland experiment. Ecology 94, 787–793. doi: 10.1890/12-1399.1

[B53] Nazarieh F. Ansari H. Ziaei A. N. Izady A. Davari K. Brunner P. (2018). Spatial and temporal dynamics of deep percolation, lag time and recharge in an irrigated semi-arid region. Hydrogeol. J. 26, 2507–2520. doi: 10.1007/s10040-018-1789-z

[B54] Peng J. Tang J. Xie S. Wang Y. Liao J. Chen C. . (2024). Evidence for the acclimation of ecosystem photosynthesis to soil moisture. Nat. Commun. 15, 9795. doi: 10.1038/s41467-024-54156-7, PMID: 39532886 PMC11557970

[B55] Peng C. C. Zeng J. Y. Chen K. S. Li Z. Ma H. L. Zhang X. . (2023). Global spatiotemporal trend of satellite-based soil moisture and its influencing factors in the early 21st century. Remote Sens. Environ. 291. doi: 10.1016/j.rse.2023.113569

[B56] Ren J. Xiao J. Ma J. He P. (2025). Quantitative assessment of the potential benefits of global afforestation on ecosystem productivity. Environ. Res. Lett. 20, 034055. doi: 10.1088/1748-9326/adbb03

[B57] Renninger H. J. Phillips N. Salvucci G. D. (2010). Wet- vs. Dry-Season Transpiration in an Amazonian Rain Forest Palm Iriartea deltoidea. Biotropica 42, 470–478. doi: 10.1111/j.1744-7429.2009.00612.x

[B58] Rodell M. Houser P. Jambor U. Gottschalck J. Mitchell K. Meng C.-J. . (2004). The global land data assimilation system. Bull. Am. Meteorol. Soc. 85, 381–394. doi: 10.1175/BAMS-85-3-381

[B59] Salley S. W. Sleezer R. O. Bergstrom R. M. Martin P. H. Kelly E. F. (2016). A long-term analysis of the historical dry boundary for the Great Plains of North America: Implications of climatic variability and climatic change on temporal and spatial patterns in soil moisture. GEODERMA 274, 104–113. doi: 10.1016/j.geoderma.2016.03.020

[B60] Seddon A. W. R. Macias-Fauria M. Long P. R. Benz D. Willis K. J. (2016). Sensitivity of global terrestrial ecosystems to climate variability. Nature 531, 229–232. doi: 10.1038/nature16986, PMID: 26886790

[B61] Seneviratne S. I. Corti T. Davin E. L. Hirschi M. Jaeger E. B. Lehner I. . (2010). Investigating soil moisture–climate interactions in a changing climate: A review. Earth-Sci. Rev. 99, 125–161. doi: 10.1016/j.earscirev.2010.02.004

[B62] Sheffield J. Wood E. F. Roderick M. L. (2012). Little change in global drought over the past 60 years. Nature 491, 435–438. doi: 10.1038/nature11575, PMID: 23151587

[B63] Shi P. Zeng J. Chen K.-S. Ma H. Bi H. Cui C. (2021). The 20-year spatiotemporal trends of remotely sensed soil moisture and vegetation and their response to climate change over the third pole. J. Hydrometeorol. 22, 2877–2896. doi: 10.1175/JHM-D-21-0077.1

[B64] Sun W. Zhou S. Yu B. Zhang Y. Keenan T. Fu B. (2025). Soil moisture-atmosphere interactions drive terrestrial carbon-water trade-offs. Commun. Earth Environ. 6, 169. doi: 10.1038/s43247-025-02145-z

[B65] Tong Y. Wang Y. Song Y. Sun H. Xu Y. (2020). Spatiotemporal variations in deep soil moisture and its response to land-use shifts in the Wind–Water Erosion Crisscross Region in the Critical Zone of the Loess Plateau (2011–2015), China. Catena 193, 104643. doi: 10.1016/j.catena.2020.104643

[B66] Traff D. C. Niemann J. D. Middlekauff S. A. Lehman B. M. (2015). Effects of woody vegetation on shallow soil moisture at a semiarid montane catchment. Ecohydrology 8, 935–947. doi: 10.1002/eco.1542

[B67] Vargas Zeppetello L. R. McColl K. A. Bernau J. A. Bowen B. B. Tang L. I. Holbrook N. M. . (2023). Apparent surface conductance sensitivity to vapour pressure deficit in the absence of plants. Nat. Water 1, 941–951. doi: 10.1038/s44221-023-00147-9

[B68] Vereecken H. Amelung W. Bauke S. L. Bogena H. Brüggemann N. Montzka C. . (2022). Soil hydrology in the Earth system. Nat. Rev. Earth Environ. 3, 573–587. doi: 10.1038/s43017-022-00324-6

[B69] Wang X. Ciais P. Wang Y. Zhu D. (2018). Divergent response of seasonally dry tropical vegetation to climatic variations in dry and wet seasons. Global Change Biol. 24, 4709–4717. doi: 10.1111/gcb.14335, PMID: 29851198

[B70] Wang L. Li X. P. Chen Y. Y. Yang K. Chen D. L. Zhou J. . (2016). Validation of the global land data assimilation system based on measurements of soil temperature profiles. Agric. For. Meteorol. 218, 288–297. doi: 10.1016/j.agrformet.2016.01.003

[B71] Wei D. Zhang Z. Yan L. Yu J. Zhang Y. Wang B. (2025). A specific time lag regulation of soil moisture across layers on soil salinization in the northeast tibetan plateau agroecosystem. Agriculture 15, 106. doi: 10.3390/agriculture15010106

[B72] Wen Y. Li M. Xu R. Qiu D. Gao P. Mu X. (2024). Spatial distribution characteristics and influencing factors of shallow and deep soil moisture under ecological restoration in the loess plateau, China. Hydrol. Proc. 38, e15109. doi: 10.1002/hyp.15109

[B73] Wu D. Wang T. Di C. Wang L. Chen X. (2020). Investigation of controls on the regional soil moisture spatiotemporal patterns across different climate zones. Sci. Total. Environ. 726, 138214. doi: 10.1016/j.scitotenv.2020.138214, PMID: 32320867

[B74] Xu L. Du H. Zhang X. (2019). Spatial distribution characteristics of soil salinity and moisture and its influence on agricultural irrigation in the ili river valley, China. Sustainability 11, 7142. doi: 10.3390/su11247142

[B75] Xu H. Lian X. Slette I. J. Yang H. Zhang Y. Chen A. . (2022). Rising ecosystem water demand exacerbates the lengthening of tropical dry seasons. Nat. Commun. 13, 4093. doi: 10.1038/s41467-022-31826-y, PMID: 35835788 PMC9283447

[B76] Xu W. Yuan W. Dong W. Xia J. Liu D. Chen Y. (2013). A meta-analysis of the response of soil moisture to experimental warming. Environ. Res. Lett. 8, 44027. doi: 10.1088/1748-9326/8/4/044027

[B77] Yang K. Zhang J. (2018). Evaluation of reanalysis datasets against observational soil temperature data over China. Climate Dynamics. 50, 317–337. doi: 10.1007/s00382-017-3610-4

[B78] Zhang Q. Fan K. K. Singh V. P. Song C. Q. Xu C. Y. Sun P. (2019). Is Himalayan-Tibetan Plateau “drying”? Historical estimations and future trends of surface soil moisture. Sci. Total. Environ. 658, 374–384. doi: 10.1016/j.scitotenv.2018.12.209, PMID: 30579195

[B79] Zhang Q. Li J. F. Gu X. H. Shi P. J. (2018). Is the Pearl River basin, China, drying or wetting? Seasonal variations, causes and implications. Global And. Planet. Change 166, 48–61. doi: 10.1016/j.gloplacha.2018.04.005

[B80] Zhao T. Dai A. (2015). The magnitude and causes of global drought changes in the twenty-first century under a low–moderate emissions scenario. J. OF. Climate 28, 4490–4512. doi: 10.1175/JCLI-D-14-00363.1

[B81] Zhou S. Williams A. P. Lintner B. R. Findell K. L. Keenan T. F. Zhang Y. . (2022). Diminishing seasonality of subtropical water availability in a warmer world dominated by soil moisture–atmosphere feedbacks. Nat. Commun. 13, 5756. doi: 10.1038/s41467-022-33473-9, PMID: 36180427 PMC9525715

[B82] Zhou S. Yu B. Schwalm C. R. Ciais P. Zhang Y. Fisher J. B. . (2017). Response of water use efficiency to global environmental change based on output from terrestrial biosphere models. Global Biogeochem. Cycles. 31, 1639–1655. doi: 10.1002/2017GB005733

