# Supplementary material for: Global soil moisture dynamics: attribution of contributions and their association with GPP
Source: Front Plant Sci. 2025 Oct 31;16:1691082. doi: 10.3389/fpls.2025.1691082 (PMC12626047; doi:10.3389/fpls.2025.1691082)
Supplement: Supplementary file 2 [file DataSheet1.docx]

Supplementary Material

## 1


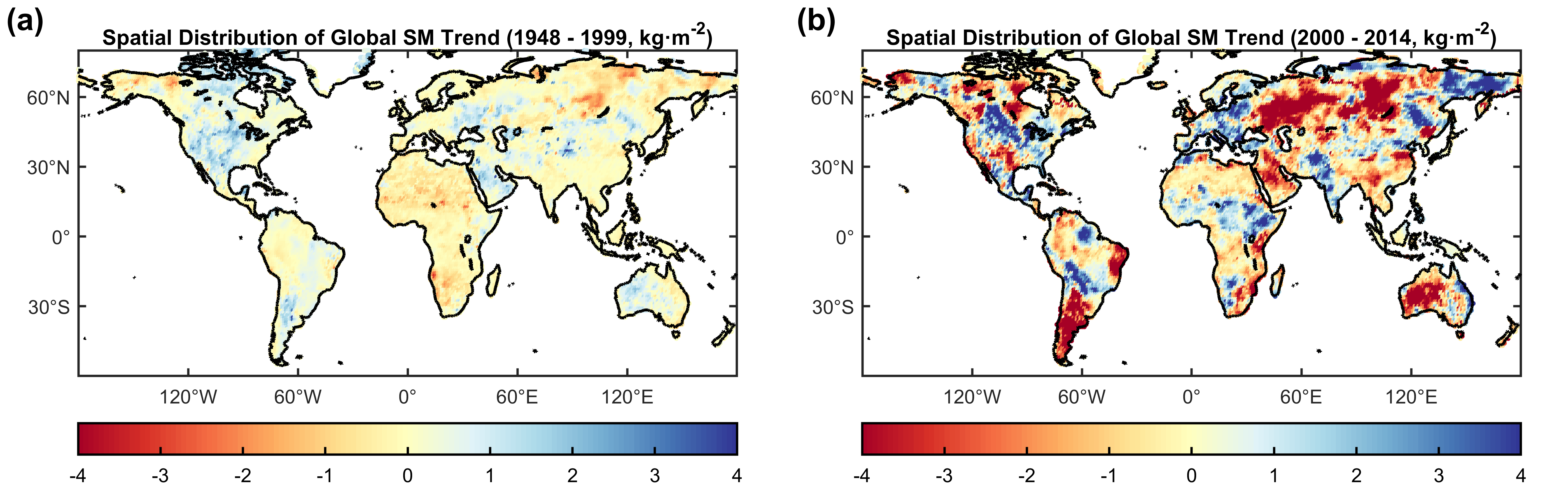


**Supplementary Figure 1.** (a) Spatial trend of global TSM, 1948–1999; (b) Spatial trend of global TSM, 2000–2014.

To validate global TSMV trends around 2000, we used the GLDAS_NOAH10_M_2.0 dataset (official record: 1948–2014, with no data beyond 2014). The series was split into 1948–1999 (52 years) and 2000–2014 (15 years) for detailed analysis. The choice of 2000–2014 as the later period reflects both dataset limits (coverage ending in 2014) and the need to focus on shifts around 2000 under consistent data sources, avoiding systematic errors from cross-dataset concatenation (e.g., switching versions after 2014).

Comparison of spatial trends across the two periods shows that, relative to 1948–1999, global TSM also declined during 2000–2014. This is consistent with findings from GLDAS_NOAH10_M_2.1 (2000–2024) reported in the main text. Despite version differences, the overlapping 2000–2014 interval produced highly consistent trend results. The two datasets provide complementary evidence—from long-term cross-period comparison (v2.0) and recent high-resolution analysis (v2.1)—jointly reinforcing the conclusion that SM has declined across most regions since 2000, thereby enhancing confidence in the findings and laying a stronger foundation for subsequent analyses of SM change mechanisms.

## 2


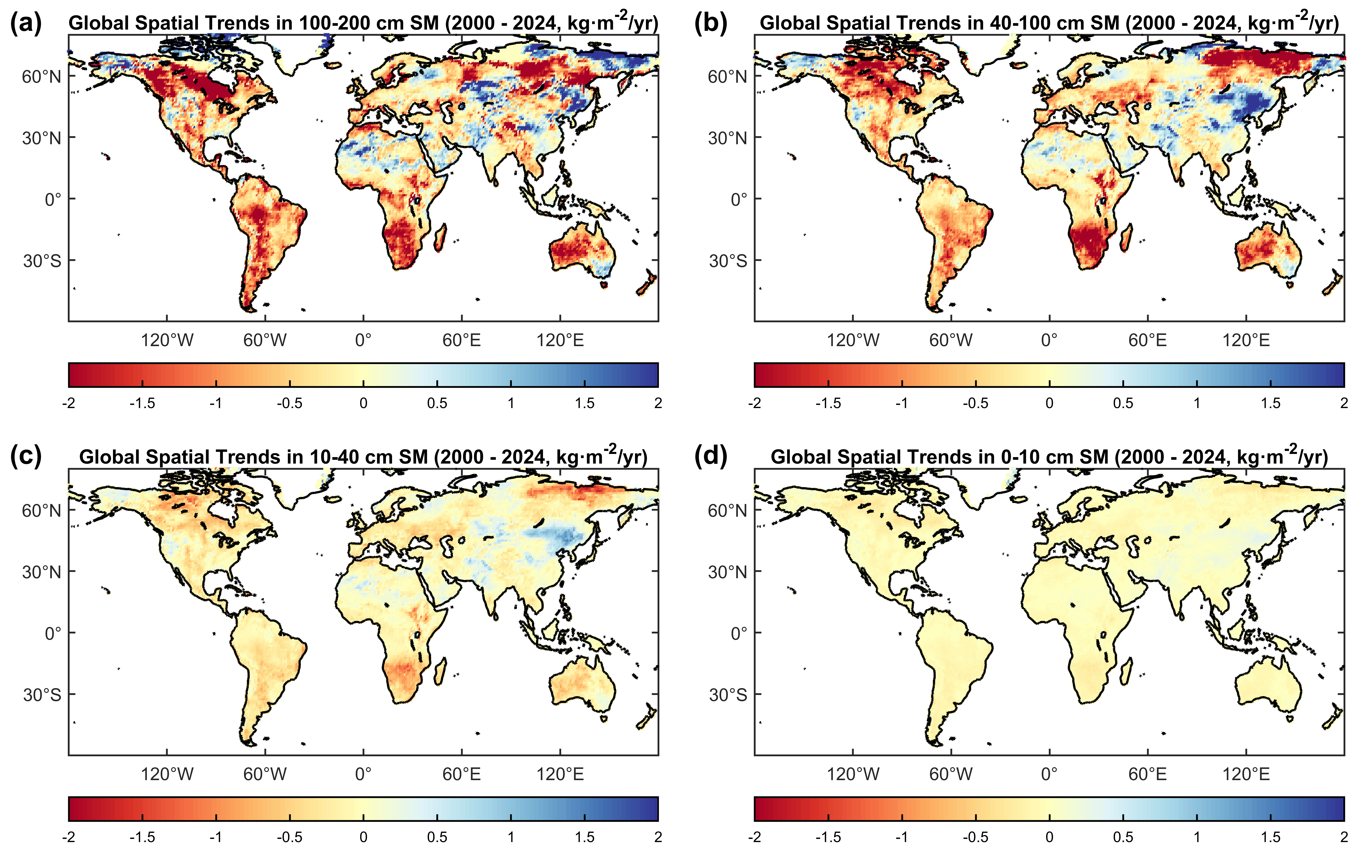


**Supplementary Figure 2.** (a–d) Spatial patterns of SM trend changes across soil depths (2000–2024).

## 3


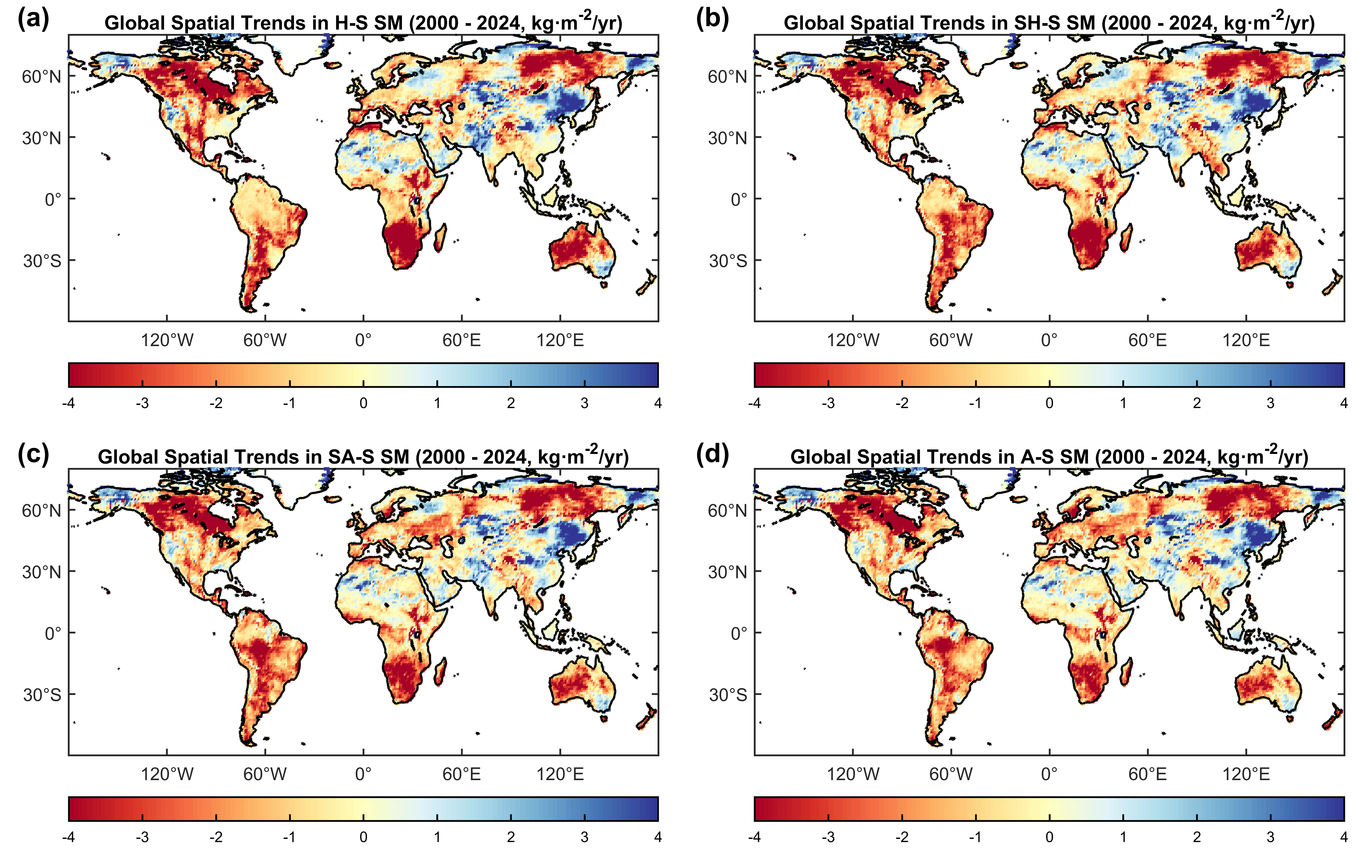

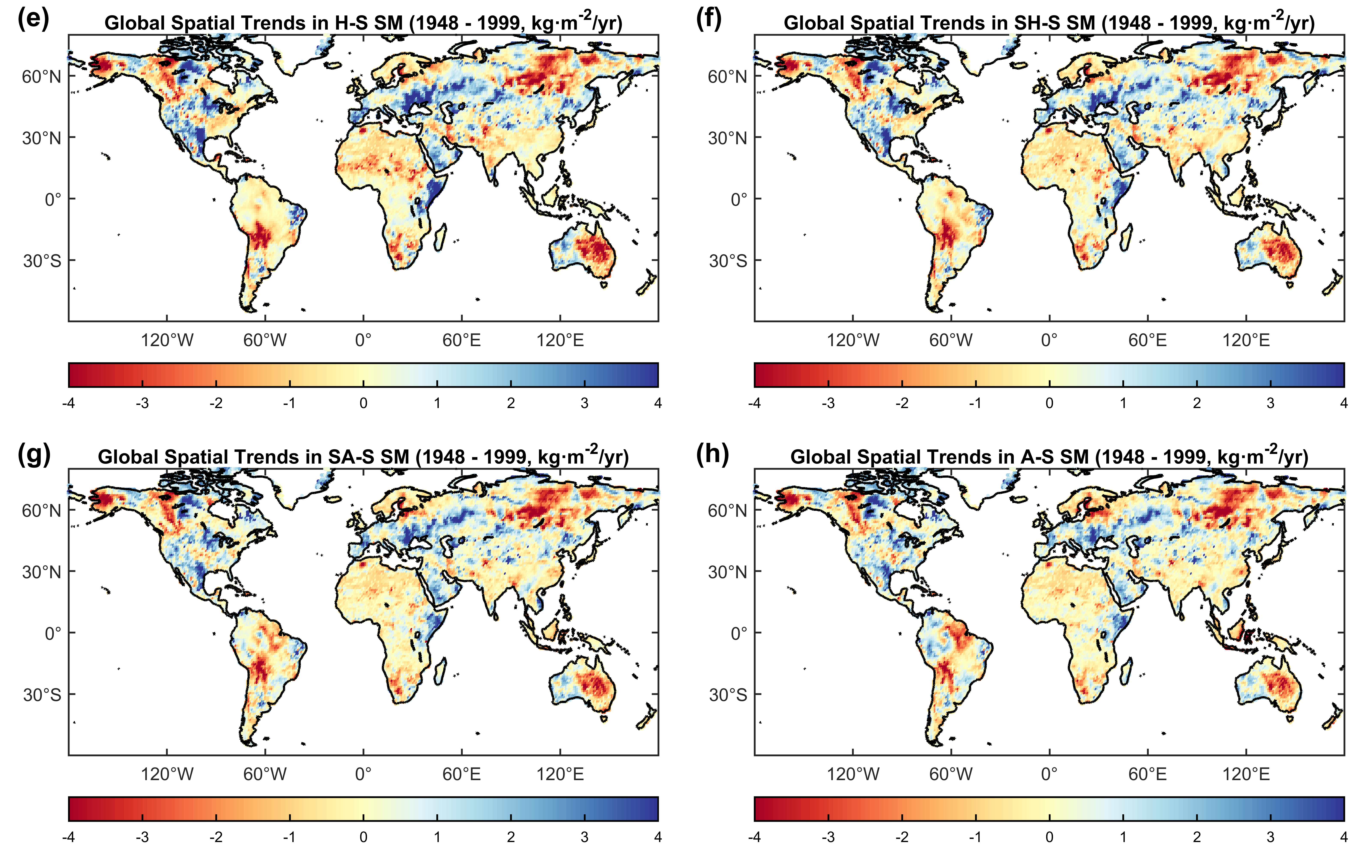


**Supplementary Figure 3.** (a–d) Spatial distributions of seasonal SM trends for 1948–1999 vs. 2000–2024.

Fig. S3 (a–h) illustrates spatial distributions of seasonal SM trends for two periods (1948–1999 and 2000–2024). During 1948–1999, SM trends across the four major seasons (A-S, SA-S, SH-S, H-S) were positive (0.0856–0.1083 kg·m⁻²·year⁻¹), indicating a slight overall increase. Since 2000, however, seasonal SM rates have turned sharply negative (−0.8149 to −0.8958 kg·m⁻²·year⁻¹), with highly consistent declines across all seasons. This shift from “slight increase” to “marked decrease” underscores the pronounced weakening of SM dynamics in the 21st century.

Comparison with Fig. 1c confirms that long-term seasonal SM time series are highly consistent with the spatial distributions in Fig. S3, all showing persistent declines. This multi-evidence convergence indicates that global SM decreases during 2000–2024 are not random fluctuations but reflect systemic long-term processes, universal across seasons.

In sum, Fig. S3 not only reveals seasonal differences in the rate shift from increasing to declining SM but also reinforces the significance of post-2000 declines across all seasons. These findings provide compelling evidence for altered seasonal water-cycle dynamics and their implications for land–atmosphere interactions.

**4**


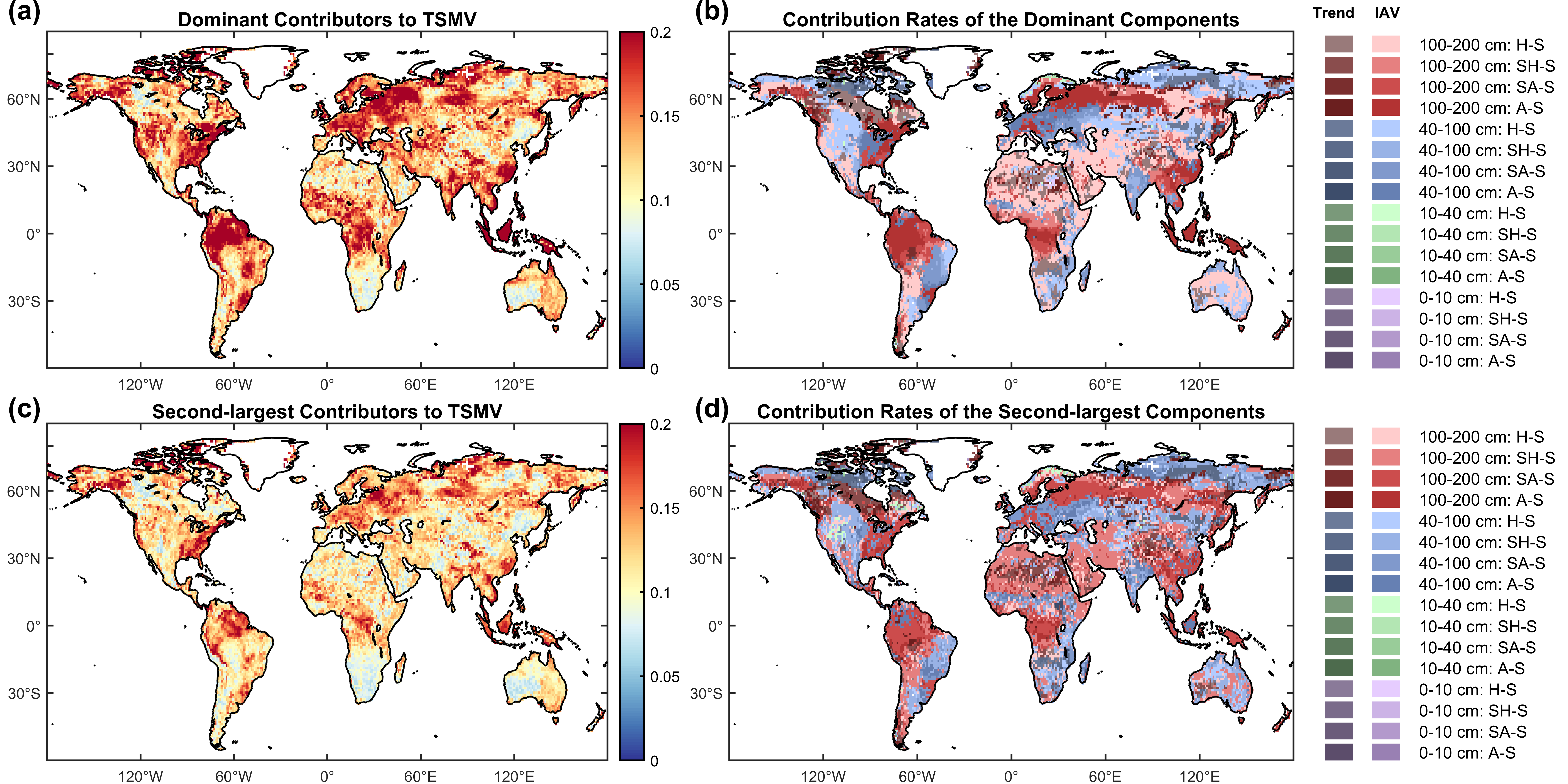


**Supplementary Figure 4.** (a–d) Dominant SM components and maximum relative contributions to TSMV across global regions (2000–2024).

Analysis of Fig. S4 (a–d) shows that IAV is the dominant driver of TSMV across all regions globally. This effect is particularly pronounced in mid–deep soils (40–200 cm), including tropical rainforests, Europe, and North America. This arises from the strong coupling between water-holding capacity at these depths and interannual rainfall variability, further reinforced by the large SM storage in these layers. In higher latitudes, processes such as freeze–thaw cycles that enhance preferential flow, soil hydrological memory, and deep-root water-use strategies collectively strengthen the role of IAV in mid–deep soils as the principal contributor to TSMV.

## 5


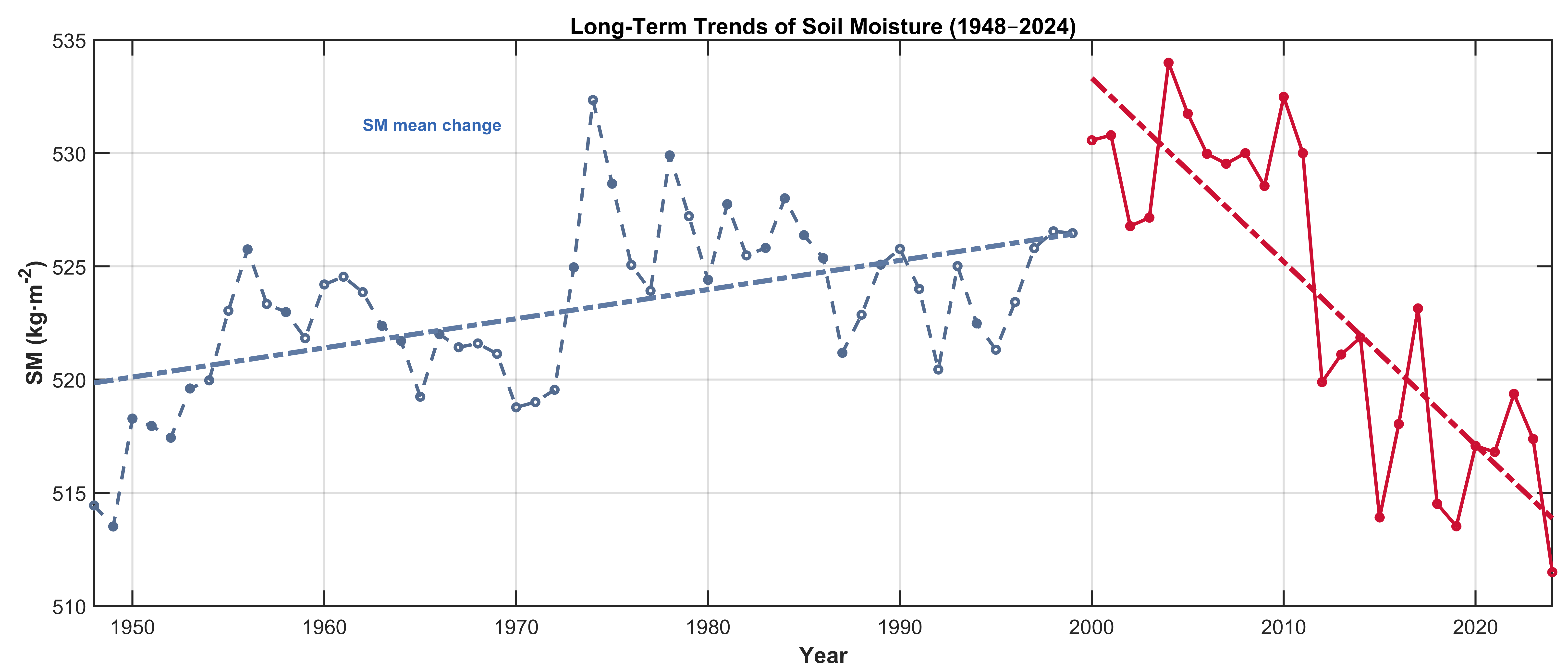


**Supplementary Figure 5.** Long-term trends in SM from 1948–2024.

As shown in Fig. S5:

1948–1999: SM exhibited a relatively stable, gradual upward Trend (blue scatter and regression line). Despite interannual variability (e.g., short-term oscillations during the 1950s–1960s), linear fitting confirms a slow cumulative increase. This reflects synergies between climate drivers (precipitation, evaporation) and land-surface factors (vegetation, land use), which tilted the “supply–consumption” balance slightly toward accumulation.

Post-2000: SM trends reversed sharply to decline (red scatter and regression line). Compared with the earlier gentle increase, the regression slope turned negative with a markedly larger magnitude, indicating accelerated SM loss. This shift suggests that since 2000, regional/global drivers—such as disrupted precipitation regimes, intensified evapotranspiration under warming, and heightened land-use intensity—have fundamentally altered the SM balance, with consumption processes dominating SM dynamics.
